# High UV and Sunlight Photocatalytic Performance of Porous ZnO Nanostructures Synthesized by a Facile and Fast Microwave Hydrothermal Method

**DOI:** 10.3390/ma14092385

**Published:** 2021-05-04

**Authors:** Sofia Henriques Ferreira, Maria Morais, Daniela Nunes, Maria João Oliveira, Ana Rovisco, Ana Pimentel, Hugo Águas, Elvira Fortunato, Rodrigo Martins

**Affiliations:** i3N/CENIMAT, Department of Materials Science, Faculty of Science and Technology, Universidade NOVA de Lisboa and CEMOP/UNINOVA, Campus de Caparica, 2829-516 Caparica, Portugal; sdl.ferreira@campus.fct.unl.pt (S.H.F.); md.morais@campus.fct.unl.pt (M.M.); daniela.gomes@fct.unl.pt (D.N.); mj.oliveira@campus.fct.unl.pt (M.J.O.); a.rovisco@campus.fct.unl.pt (A.R.); acgp@campus.fct.unl.pt (A.P.); hma@fct.unl.pt (H.Á.)

**Keywords:** ZnO, porous nanostructures, zinc hydroxide carbonate, hydrothermal synthesis, microwave, sunlight photocatalysis

## Abstract

The degradation of organic pollutants in wastewaters assisted by oxide semiconductor nanostructures has been the focus of many research groups over the last decades, along with the synthesis of these nanomaterials by simple, eco-friendly, fast, and cost-effective processes. In this work, porous zinc oxide (ZnO) nanostructures were successfully synthesized via a microwave hydrothermal process. A layered zinc hydroxide carbonate (LZHC) precursor was obtained after 15 min of synthesis and submitted to different calcination temperatures to convert it into porous ZnO nanostructures. The influence of the calcination temperature (300, 500, and 700 °C) on the morphological, structural, and optical properties of the ZnO nanostructureswas investigated. All ZnO samples were tested as photocatalysts in the degradation of rhodamine B (RhB) under UV irradiation and natural sunlight. All samples showed enhanced photocatalytic activity under both light sources, with RhB being practically degraded within 60 min in both situations. The porous ZnO obtained at 700 °C showed the greatest photocatalytic activity due to its high crystallinity, with a degradation rate of 0.091 and 0.084 min^−1^ for UV light and sunlight, respectively. These results are a very important step towards the use of oxide semiconductors in the degradation of water pollutants mediated by natural sunlight.

## 1. Introduction

The degradation of organic pollutants present in wastewater has developed into a topic of great importance, and has become the focus of many research fields. The organic dyes and compounds used in various industries (such as paper, textile, cosmetic, pharmaceutical, and plastics) lead to contaminated wastewaters that may have harmful effects in aqueous ecosystems and, therefore, on human health [1,2,3]. As such, many researchers have been looking to low-cost semiconductor oxides for the degradation of these pollutants through photocatalysis treatment processes.

Zinc oxide (ZnO) has emerged as a promising photocatalyst due to its unique properties, such as its direct and wide bandgap of 3.37 eV and its high exciton binding energy of 60 meV, as well as its nontoxicity, chemical stability, abundance in nature, and low cost [4]. However, ZnO is limited in its use as a photocatalyst due to the fast recombination rate of photogenerated carriers, its very poor response to visible light, and photocorrosion in aqueous solution under UV irradiation [5,6]. Nonetheless, there are different methods to improve the photocatalytic efficiency of ZnO (mainly under visible light [7]), including changing the ZnO morphology at the nanoscale [8,9], introducing/controlling bulk and surface defects [10,11], coupling ZnO with other materials [12,13,14,15], and doping with metal or nonmetal ions [16,17,18]. However, it is not an easy task to compare the photocatalytic performance between works using different ZnO materials, since the experimental setup differs greatly from work to work [19,20,21,22,23]. Experimental parameters, such as irradiation light intensity and wavelength, photocatalyst properties and amounts, pollutant species or models, pollutant concentrations and solution volumes, and the relative position and configuration of the photocatalytic reactor to the light source have a great influence on the photocatalytic performance of the tested nanomaterials [24]. Therefore, it would only be possible to correctly compare ZnO nanomaterials as photocatalysts among works wherein similar experimental conditions are employed.

ZnO nanostructures can be produced through a variety of synthesis methods, such as electrodeposition [25], chemical bath deposition [26], chemical vapor deposition [27], and solvothermal [28] or hydrothermal [29,30] synthesis, either by conventional or microwave-assisted heating [31,32,33,34,35]. Microwave-assisted hydrothermal/solvothermal synthesis represents an attractive synthesis method when compared to conventional heating due to its high reaction rate, since microwave radiation is absorbed by the species present in the reaction medium, which results in a homogeneous and rapid volumetric heating [35,36,37]. Depending on the precursors used and their concentrations, the selected solvents, and the pH of the solution, ZnO can be easily synthesized into several morphologies with high specific surface areas, such as nanorods, quantum dots, nanoplates, nanoneedles, nanotubes, nanoflowers, nanobelts, and nanowires [38,39,40,41,42,43].

Among ZnO nanomaterials, porous nanostructures with nanoscale thicknesses have received special attention due to their large specific surface area, which can enhance their performance in numerous applications [44,45,46,47,48,49]. Porous ZnO nanostructures can be obtained by the thermal decomposition of layered zinc hydroxide (LZH) materials [20]. LZHs are composed of positively charged zinc hydroxyl layers intercalated by anions and water molecules [50]. In particular, LZHs intercalated by carbonate ions (LZHC) are a promising precursor material to obtain porous ZnO nanostructures [20,51,52,53,54]. LZHC is typically obtained by hydrothermal methods with the desired morphology and then converted into porous ZnO nanostructures via calcination at high temperatures [55]. During thermal decomposition, the LZHC precursor releases gaseous molecules and, consequently, the original structure contracts and pores are formed throughout the materials [20]. In a previous work from our group [56], the application of porous ZnO nanostructures in nanogenerators via microwave assisted hydrothermal synthesis and calcination of a LZHC precursor was demonstrated.

In this work, porous ZnO nanostructures assembled into 3D hierarchical structures were obtained following the same procedure as in our previous work [56]. However, in this study, the LZHC precursor was calcinated at different temperatures and the influence of the calcination temperature on the final products was investigated. The produced porous ZnO nanostructures were tested as photocatalysts in the degradation of rhodamine B (RhB) under UV and sunlight irradiation. Other authors have also studied the application of 3D hierarchical ZnO structures for the degradation of RhB under UV light and/or solar light [19,20,22,23,57]. However, the reported works typically employ conventional hydrothermal methods that take long periods of time and dope ZnO with other elements to increase its photocatalytic efficiency. For instance, Haibo et al. [23] synthesized carbon-doped ZnO foam-like 3D structures via a hydrothermal method and tested them under natural sunlight. These authors obtained 98% RhB degradation after 25 min using a catalyst concentration of 1 g L^−1^ and a RhB solution concentration of 5 mg L^−1^. Liu et al. [20] have also doped porous ZnO nanostructures with carbon by hydrothermal method for the photodegradation of RhB under visible light (λ > 420 nm), obtaining > 90% RhB degradation after 80 min using 5 g of catalyst per liter of RhB solution (10 mg L^−1^).

To the best of our knowledge, this is the first work to report the high photocatalytic efficiency of porous ZnO nanostructures synthesized by microwave hydrothermal method under UV and natural sunlight irradiation, where RhB was almost completely degraded within only 60 min of UV and sunlight exposure. These results are of great significance, as they demonstrate (along with our previous work [56]) the multifunctionality of porous ZnO nanostructures synthesized by a facile, fast, green, and low-cost hydrothermal method assisted by microwave irradiation.

## 2. Materials and Methods

### 2.1. Synthesis and Characterization of Porous ZnO Nanostructures

As reported in our previous work [56], the fabrication process of porous ZnO nanostructures can be divided into two steps. Firstly, a LZHC precursor is synthesized via hydrothermal method assisted by microwave irradiation. Secondly, the LZHC is calcinated at high temperatures where it turns into porous ZnO nanostructures. Briefly, for the synthesis of LZHC, an aqueous solution with 0.05 M of zinc nitrate hexahydrate (Zn(NO_3_)_2_·6H_2_O, Sigma–Aldrich 98%, Sigma–Aldrich, St. Louis, MO, USA) and 0.25 M of urea (CH_4_N_2_O, Sigma–Aldrich 99.0–100.5%, Sigma–Aldrich, St. Louis, MO, USA) was prepared. The synthesis was carried out in a CEM Discovery SP microwave (CEM, Matthews, NC, USA) at 140 °C for 15 min under a power of 100 W. The resulting LZHC powder was washed with deionized water and isopropanol and dried in air at room temperature for 48 h. The dried LZHC was then calcinated in air in a muffle furnace (Nabertherm GmbH, Lilienthal, Germany) at 300, 500, and 700 °C for 2 h with a heating rate of 250 °C h^−1^.

X-ray diffraction (XRD) was carried out to evaluate the crystallinity of the produced ZnO nanostructures using a PANalytical’s X’Pert PRO MRD X-ray diffractometer (PANalytical B.V., Almero, The Netherlands), with a monochromatic Cu Kα radiation source with a wavelength of 1.540598 Å. XRD measurements were performed with a scanning step size of 0.016 ° from 10 to 90 ° (2θ), with a scanning step size of 0.016 °. Scanning electron microscopy (SEM) was used to evaluate the morphology of the calcinated ZnO products in a Carl Zeiss AURIGA CrossBeam FIB-SEM workstation equipped with an Oxford X-ray Energy Dispersive Spectrometer (Carl Zeiss Microscopy GmbH, Oberkochen, Germany). The apparent specific surface area was determined by employing the Brunauer−Emmet−Teller (BET) method to the adsorption isotherms obtained from nitrogen gas adsorption at 77 K in an adsorption apparatus (ASAP 2010 Micromeritics, Norcross, GA, USA), where the samples were degassed beforehand at 150 °C in vacuum.

Raman spectroscopy measurements of the calcinated ZnO products were performed in a Renishaw inVia Reflex micro-Raman spectrometer (Renishaw plc, Wotton-under-Edge, UK) equipped with an air-cooled CCD detector and a HeNe laser operating at 50 mW of 532 nm laser excitation. The spectral resolution of the spectroscopic system was 0.3 cm^−1^. The laser beam was focused with a 50× Leica objective lens (N Plan EPI, Leica Microsystems, Wetzlar, Germany) with a numerical aperture of 0.75. An integration time of two scans (30 s each) was used for all measurements to reduce the random background noise induced by the detector without significantly increasing the acquisition time. The intensity of the incident laser was 0.5 mW. All spectra were obtained in triplicate for each sample at room temperature in the wavelength range between 70 and 17,800 nm. Triplicates of all samples were performed. All the raw data were collected digitally with Wire 5.0 software for processing, including cosmic ray removal, noise reduction, and baseline. Fitting with Lorentzian was performed in order to identify the different vibrational bands. Fourier transform infrared (FTIR) spectroscopy measurements were carried out using an attenuated total reflectance (ATR) sampling accessory (Smart iTR, ThermoFisher Scientific Inc., Waltham, MA, USA) equipped with a single bounce diamond crystal on a Thermo Nicolet 6700 spectrometer (ThermoFisher Scientific Inc., Waltham, MA, USA). The FTIR spectra were acquired with a 45 ° incident angle in the range between 4000 and 500 cm^−1^ and with a 4 cm^−1^ resolution.

Diffuse reflectance measurements of the porous ZnO nanostructures were carried out using a PerkinElmer lambda 950 UV/VIS/NIR spectrophotometer (PerkinElmer, Waltham, MA, USA) with an integrating sphere with a 150 mm diameter internally coated with Spectralon. The reflectance spectra were obtained at room temperature from 350 to 800 nm. A standard Spectralon reflector sample was used as a reference (R = 1.0) to calibrate the system.

The photoluminescence (PL) of the porous ZnO structures was evaluated using a PerkinElmer LS55 luminescence spectrometer (PerkinElmer, Waltham, MA, USA) equipped with a Xenon lamp as an excitation source. The PL measurements were performed at room temperature, from 350 to 700 nm, using an excitation wavelength of 254 nm and a cut-off filter of 350 nm.

### 2.2. Characterization of Porous ZnO Nanostructures as Photocatalysts

The photocatalytic activity of the porous ZnO nanostructures calcinated at different temperatures under UV radiation was characterized by the degradation of rhodamine B (RhB) from Sigma–Aldrich, at room temperature. For this purpose, 25 mg of each sample were dispersed into 50 mL of RhB aqueous solution (4 mg L^−1^) and then stirred for 30 min in the dark to establish adsorption–desorption equilibrium. The solutions with the dispersed ZnO nanostructures were then exposed to UV irradiation using three mercury lamps (model HNSL from Osram Puritec) with a power of 95 W each, a wavelength of 254 nm, and a total UV light intensity of 35 mW cm^−2^. The solution vessels were placed at 27.5 cm from the UV light source. The absorbance spectra of the solutions were recorded using a PerkinElmer lambda 950 UV/VIS/NIR spectrophotometer with intervals of 15 min until 60 min of UV exposure to investigate the photocatalytic performance of the produced samples on the degradation of RhB. After each UV exposure, 4 mL of the RhB solutions with the dispersed nanostructures were collected after centrifugation for the absorbance measurement and then returned to the container for further UV exposition. The porous ZnO nanostructures were also tested as photocatalysts under natural sunlight around midday for a total exposure time of 60 min. Sunlight intensity was found to be around 89 mW cm^−2^ (0.89 sun) during the photocatalysis experiments using a solar power meter from Sciencetech (Sciencetech-Inc, London, ON, Canada). Here, the absorbance spectra of the aqueous RhB solutions with the dispersed photocatalysts were recorded with intervals of 15 min until 60 min of sunlight exposure. As before, after each sunlight exposure, 4 mL of the tested solutions were collected after centrifugation for the absorbance measurement and then returned to the container for further sunlight exposure.

## 3. Results and Discussion

### 3.1. Characterization of the Porous ZnO Nanostructures

**Figure 1:**
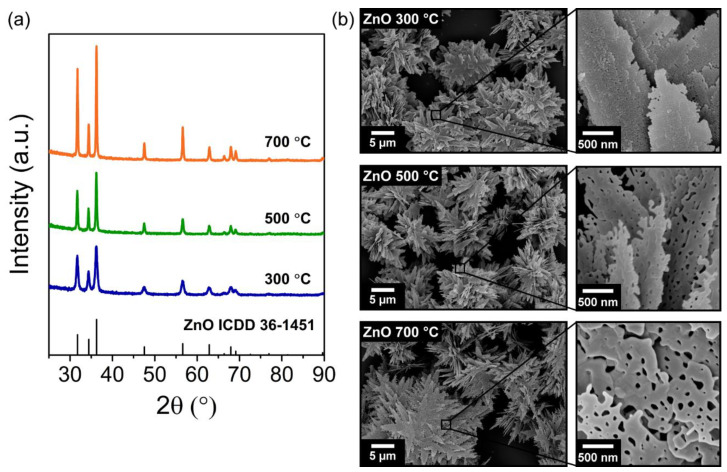
(**a**) XRD diffractograms and (**b**) SEM images of porous ZnO nanostructures synthesized via the hydrothermal method (assisted by microwave irradiation) and calcinated at 300, 500, and 700 °C.

Porous ZnO nanostructures were produced via the calcination of a LZHC precursor. LZHC was synthesized by hydrothermal method assisted by microwave irradiation and it was fully characterized in our previous report [56]. After the calcination process at 300, 500, and 700 °C, the LZHC precursor was successfully converted into porous ZnO nanostructures, which can be inferred from the X-ray diffractograms of the obtained samples, shown in Figure 1a. All the peaks in the diffractograms correspond to the hexagonal wurtzite ZnO structure (ICDD 36-1451). No characteristic peaks from any other impurities were detected, indicating that the LZHC precursor was completely converted into ZnO. Additionally, the diffraction peaks become narrower with increasing calcination temperature, leading to an increase in the crystallite size of the ZnO samples. SEM images of the porous ZnO products are presented in Figure 1b with different magnifications. The low magnification images show that the ZnO nanomaterials are assembled to hierarchically form flower-like 3D structures, as observed in our previous work [56]. However, when observing the high magnification SEM images, it is possible to see that the ZnO nanoplates present a porous structure with serrate edges, where higher calcination temperatures result in larger pores with a wider size distribution.

The porous ZnO nanostructures were further investigated by N_2_ adsorption/desorption measurements. The resulting adsorption isotherm curves are presented in Figure S1. The obtained isotherm curves and hysteresis loop can be ascribed to type IV, according to the IUPAC classification [58], which indicates the presence of mesopores (2 to 50 nm in size) [20]. When the calcination temperature increases, the hysteresis loop shifts to higher relative pressures and the loop area decreases, resulting in smaller specific surface areas and larger pores [20,58]. Using Brunauer–Emmett–Teller (BET) analysis, it was found that ZnO nanostructures calcinated at 300 °C have the highest specific surface area of 38.51 m^2^ g^−1^, while calcination temperatures of 500 and 700 °C resulted in specific surface areas of 11.68 and 6.41 m^2^ g^−1^, respectively. From the SEM images in Figure 1b, it appears that the 300 °C calcination temperature originates smaller mesopores in higher concentration and narrower size distribution. However, with increasing temperature, the smaller pores start to coalesce into larger ones, which leads to ZnO nanoplates with larger mesopores with a higher size distribution.

**Figure 2:**
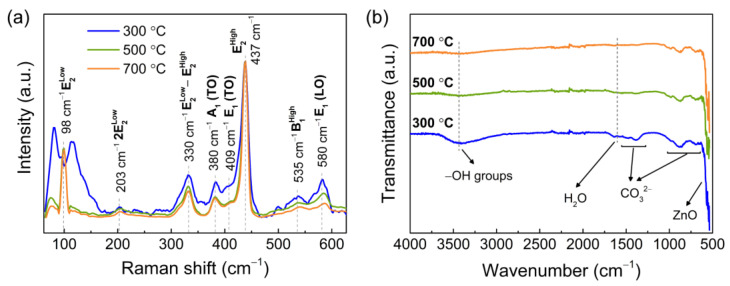
(**a**) Raman spectra and (**b**) FTIR spectra of porous ZnO nanostructures calcinated at 300, 500, and 700 °C.

The normalized Raman spectra of the porous ZnO nanostructures are shown in Figure 2a. In ZnO, the lattice optical phonons at the point Γ of the Brillouine zone is given by the optical symmetry modes A_1_ + 2B_1_ + E_1_ + 2E_2_ [31,59]. The active Raman modes include the modes A_1_, E_1_, and E_2_, with A_1_ and E_1_ also being infrared active and splitting into longitudinal–optical (LO) and transversal–optical (TO) components [60]. Based on the Raman spectra in Figure 2a, the two most intense peaks are located at 98 and 437 cm^−1^ and are attributed to the E_2_^High^ and E_2_^Low^ optical modes, respectively, which are characteristic of the wurtzite hexagonal of ZnO [61]. The peak at 380 cm^−1^ is associated with the A_1_ (TO) mode, while the peak at 409 cm^−1^ is related to the E_1_ (TO) mode. The band around 580 cm^−1^ is reported to be a superposition of the A_1_ (LO) mode at 574 cm^−1^ and the E_1_ (LO) mode at 583 cm^−1^ [62]. The porous ZnO nanostructures calcinated at 300 °C show an intense band at low wavenumbers, with two prominent peaks at 80 and 115 cm^−1^. These two peaks are present in the Raman spectrum of the LZHC precursor shown in Figure S2, suggesting an incomplete pyrolysis of the precursor into ZnO for calcination temperatures of 300 °C. Higher calcination temperatures result in more defined and narrower peaks, which are an indicator of samples with higher crystallinity [63,64], as corroborated by the X-ray diffractograms in Figure 1a. Moreover, Y. Song et al. [65] demonstrated that the absorption band from around 80 to 190 cm^−1^ may be related to interstitial zinc defects, whereas the intensity of the band around 580 cm^−1^ may be attributed to oxygen vacancies. In addition, the band around 535 cm^−1^ in the Raman spectra has been assigned to B_1_^high^ mode. Although this mode is Raman inactive, its appearance may occur due to modification of the translational crystal symmetry induced by defects or impurities in the ZnO structure [59,66].

The FTIR spectra of the porous ZnO nanostructures calcinated at different temperatures are displayed in Figure 2b. The strong absorbance band from 632 cm^−1^ to lower wavenumbers is characteristic of wurtzite ZnO, which usually presents a strong infrared peak around 443 cm^−1^ that cannot be detected with the used FTIR spectrometer. This absorption region is assigned to the stretching vibration mode of Zn–O [67,68,69]. The bands from 1570 to 1390 cm^−1^ as well as the bands from 1100 to 700 cm^−1^ are attributed to the presence of CO_3_^2−^ from the LZCH precursor [70,71,72]. The small absorption band found at 1640 cm^−1^ is associated with the bending mode of the water molecule [73,74]. The broad band around 3400 cm^−1^ is attributed to the vibration mode of hydroxyl groups (–OH), which are known to be actively adsorptive sites [75]. However, this broad band decreases with calcination temperature, alongside the CO_3_^2−^ absorption bands, which result from the incomplete pyrolysis of the LZHC precursor. As such, as shown in Raman spectra and the X-ray diffractograms, a calcination temperature of 700 °C results in purer and more crystalline ZnO nanostructures.

**Figure 3:**
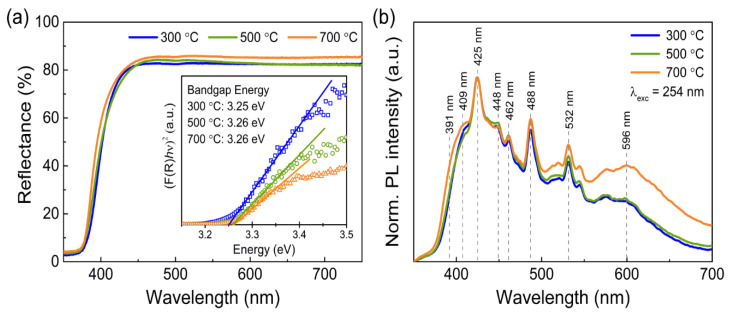
(**a**) Reflectance spectra of the porous ZnO nanostructures calcinated at 300, 500, and 700 °C with an inset graphic showing the obtained bandgap energy for each calcination temperature through the K-M function. (**b**) Photoluminescence spectra of the porous ZnO samples measured with an excitation wavelength of 254 nm.

The UV–Vis diffuse reflectance of the produced ZnO samples is presented in Figure 3b. A slight blue shift in the absorption edge is observed for higher calcination temperatures. This shift occurs due to the increase in the crystallinity of ZnO with increasing temperature, as shown in Figure 1a.

As shown through Raman and FTIR analyses, the presence of bulk defects is more significant in the porous ZnO nanostructures calcinated at 300 and 500 °C. These defects create extra levels between the valence and conduction band [19,76] that lead to a shift of the absorption edge to longer wavelengths and increase the absorption ability in the visible range, indicating that these nanostructures may have a good photocatalytic performance under visible light [77].

The optical band gap *E_g_* was calculated by applying the Kubelka–Munk (K-M) method to the reflectance (*R*) data [76]. The K-M method is based on the following equation:

(1) $$F\left(R\right)=\frac{{\left(1-R\right)}^{2}}{2R}$$

The K-M function (*F(R)*) is proportional to the absorption coefficient (*α*). Therefore, by considering the Tauc relation, the following expressions can be obtained [78]:

(2) $$F\left(R\right)\propto \;\alpha \;\propto \frac{{\left(h\upsilon -E_{g}\right)}^{1/n}}{h\upsilon }$$

(3) $${\left(F\left(R\right)h\upsilon \right)}^{n}=A\left(h\upsilon -E_{g}\right)$$

where *A* is a constant and *n* is equal to 2 for semiconductors with direct allowed transitions [79]. As shown by the inset graph in Figure 3a, the value of *E_g_* can be determined by extrapolating the linear part of the function curve with the energy axis. The estimated bandgap energies are 3.25, 3.26, and 3.26 eV for the ZnO nanostructures obtained at 300, 500, and 700 °C, respectively, which is consistent with the observed slight shift in the absorption edge and with the values reported in the literature [20,77].

The photoluminescence (PL) spectra of the porous ZnO nanostructures are presented in Figure 3b. Although the origin of PL in ZnO is typically assigned to the recombination of the photoinduced carriers and the intrinsic defects of this metal oxide’s nanostructures, a consensus concerning the exact origin of the defect-related visible PL of ZnO has not yet been reached. Indeed, numerous hypotheses have been proposed to explain the emission in this region over the last decades [62,80,81,82,83,84]. Nevertheless, it is commonly accepted that the nature of visible PL in ZnO revolves around intrinsic defects in the ZnO matrix, such as interstitial zinc and oxygen (Zn_i_ and O_i_), antisite oxygen (O_Zn_), and oxygen and zinc vacancies (V_O_ and V_Zn_) [85]. In micro- and nanostructured ZnO, the PL emission is widely influenced by the surface and interface properties and, therefore, the synthesis method plays an important role in the resulting PL signal [86,87,88,89]. If the surface-to-volume ratio of ZnO nanostructures is high, then the PL spectra will mostly be influenced by surface-related defects, where new emission bands may arise and dominate over the bulk-related luminescence [81]. Therefore, by controlling the distribution of surface vs bulk defects, it is possible to tailor the material’s PL properties.

The normalized PL spectra of the porous ZnO nanostructures, shown in Figure 3b, depict several emission bands in the blue (400–490 nm), green (500–550 nm), and yellow–orange (~600 nm) regions of the spectra. From Figure 3b, it is evident that the blue emission bands are more intense than the green and yellow–orange bands. Figure S3a in Supplementary Materials shows the deconvolution of one of the spectra into several Gaussian components, and the values of the main Gaussian peaks are marked in Figure 3b. The component centered around 391 nm is usually attributed to the recombination of free excitons formed upon excitation [80]. ZnO has a free binding energy (*E_X_*) of around 60 meV [59,90] and, therefore, the energy of the radiative recombination would be equal to *E_g_-E_X_*. By taking into account the *E_g_* value calculated for ZnO (~3.25–3.26 eV), this emission would have a value around 3.2 eV (~388 nm), which is close to the observed emission band at 391 nm. The blue emissions between 409 and 488 nm have been assigned by several authors to the transitions between Zn_i_ and extended Zn_i_ levels and the valence band (VB) [59,62,80,84,89]. Zn_i_ defects form a shallow donor level and many PL studies have considered the location of this level at 0.22 eV below the conduction band (CB) [84,89,91,92]. The transition from this level to the VB would originate an emission at around 410 nm, which is in good accordance with the observed band at 409 nm in Figure 3b. In particular, Zeng et al. [84] demonstrated that the violet and blue emissions between 415 and 488 nm of ZnO nanoparticles originate from transitions from Zn_i_ and extended Zn_i_ levels to the VB, which is in good agreement with the observed emission bands in the same region in this work.

The green PL emission observed around 532 nm has been associated with different defects in ZnO and, as such, it is considered one of the most controversial emission bands of ZnO. Many authors have attributed this emission to the transition between the CB and Zn_i_ levels to the V_O_ defect level [59,62,91,93,94,95,96], whereas other works suggest that it is in fact the transitions involving V_Zn_ defects that generate the green PL in ZnO [83,97,98,99,100]. Despite the lack of consensus about the origin of the green emission, it has been demonstrated by many groups that this emission is mostly governed by surface defects [62,86,88,101], meaning that ZnO nanostructures with high specific surface areas will most likely present strong emissions in this region, as was observed for the porous ZnO nanostructures in this work.

Regarding the yellow emission observed around 596 nm, it has been reported that it may originate from the transition from the CB or Zn_i_ to the O_i_ defect level, which has been placed at around 2.28 eV below the CB [81,86,88,102,103,104], which is in good agreement with the yellow–orange PL in this work. It has been suggested that annealing at high temperatures in an oxygen-rich environment increases the intensity of this emission [86,103], which may explain the higher intensity of this PL band for the porous ZnO nanostructures calcinated at 700 °C in air.

In summary, it should be noted that the PL emission usually observed for ZnO is composed of several emission bands, as suggested in Figure S3a, which indicates that distinct transitions involving different defect levels may contribute to the PL emission in the same region of the spectrum. Therefore, a straightforward PL analysis is not possible in most cases and complementary characterization techniques are needed to infer the origin of each observed PL band. In spite of this, the PL signal acquired in this work for all of the produced porous ZnO nanostructures indicates that these materials can be excited with sub bandgap excitation energies, as proven by the PL spectra in Figure S3b, where the ZnO obtained at 700 °C presented visible PL emissions for different excitation wavelengths. These results show that these nanostructures can absorb light in the visible range and, therefore, they can be applied in visible light photocatalysis.

### 3.2. Photocatalytic Activity of the Porous ZnO Nanostructures

**Figure 4:**
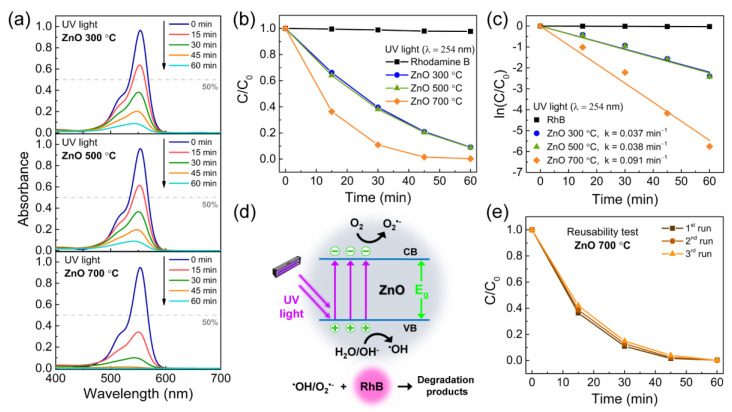
(**a**) Absorbance spectra of RhB recorded at different degradation times under UV irradiation for ZnO samples calcinated at 300, 500, and 700 °C. (**b**) Degradation ratio *C/C*_0_ of RhB vs. UV exposure time for all the produced porous ZnO samples. (**c**) ln(*C/C*_0_) vs. UV exposure time and the resultant degradation rates *k* for each tested sample. (**d**) UV light photocatalytic mechanism of ZnO. (**e**) Reusability tests of the ZnO sample calcinated at 700 °C showing the degradation ratio *C/C*_0_ for three photocatalytic runs.

Rhodamine B is commonly used as a model dye contaminants in photocatalytic tests [24,105,106,107,108,109]. RhB is highly soluble in water and has an absorption peak in the visible range around 554 nm, meaning that its degradation can be easily monitored using optical absorbance spectroscopy. As such, the UV photocatalytic activity of the porous ZnO nanostructures was evaluated from the intensity decrease of the maximum absorption peak of the RhB solution after UV light exposure. The absorbance spectra of RhB recorded at different degradation times for ZnO samples calcinated at 300, 500, and 700 °C are presented in Figure 4a. Based on these absorbance spectra, it can be observed that all tested calcination temperatures yielded very good degradation values after only 60 min of UV light exposure. Figure 4b shows the ratio between the absorbance value at each exposure time, *C*, and the initial absorbance of the RhB solution, *C*_0_. The degradation percentage can then be calculated using

(4) $$Degradation\;\left(\%\right)=\frac{\left(C_{0}-C\right)}{C_{0}}\times 100$$

The degradation percentage was found to increase with the calcination temperature, presenting values around 91%, 92%, and >99% for ZnO nanostructures calcinated at 300, 500, and 700 °C, respectively. In addition to the visible absorbance spectra of RhB, the efficiency of the photocatalytic process can also be evaluated by the constant rate *k* of the process. This constant is given by the following pseudo first-order kinetics reaction equation, valid for pollutant concentrations in the millimolar range [110]:

(5) $$\ln\frac{C}{C_{0}}=kt$$

Figure 4c shows the ln(*C*/*C*_0_) curves of the RhB solution without and with the ZnO nanostructures for each UV exposure time. All the presented curves were linearly fitted to obtain the constant rate *k* of the degradation process. ZnO nanostructures calcinated at 700 °C present a degradation rate around 0.091 min^−1^, whereas calcination temperatures of 300 and 500 °C result in similar degradation rates: 0.037 and 0.038 min^−1^, respectively.

The UV photocatalytic mechanism (Figure 4d) is based on the photogeneration of electron-hole (e^−^/h^+^) pairs in the semiconductor photocatalyst when exposed to UV light with energy close to that of the semiconductor bandgap. The photoinduced e^−^/h^+^ pairs can recombine or migrate to the semiconductor surface and participate in a series of oxidation–reduction reactions with adsorbed species. The photogenerated holes in the valence band can react with surface-bound hydroxyl groups (–OH) and adsorbed water molecules to form hydroxyl radicals (^•^OH) [111]. On the other hand, electrons in the conduction band react with oxygen molecules to form superoxide radicals (O_2_^•−^) [112]. The O_2_^•−^ species contribute to the formation of hydrogen peroxide that, together with ^•^OH radicals, leads to the degradation of the dyes [113].

There are several factors that influence the photocatalytic efficiency of semiconductors materials, including specific surface area, bandgap energy, morphology, crystallinity and crystal facets, surface and bulk defects, and nanoparticle size [114,115]. Even though the specific surface area plays an important role in the photocatalytic performance of nanostructures due to the large number of active adsorption sites, in the UV light experiments performed in this work high specific surface areas do not necessarily mean high photocatalytic efficiencies. In fact, the porous ZnO nanostructures calcinated at 700 °C show the highest degradation rate in spite of being the nanostructures with the lowest specific surface area. In this case, the high crystallinity of these ZnO nanostructures, as observed in Figure 1a, is the most critical factor influencing their photocatalytic activity. High crystallinities indicate a decrease in the relative concentration of bulk to surface defects [116]. Surface defects not only serve as adsorption sites but also as charge carrier traps, where the trapped carriers can then be transferred to the adsorbed species, which prevents the recombination of photogenerated e^−^/h^+^ pairs [19,117,118]. On the other hand, bulk defects can act as recombination centers for the photogenerated carriers, leading to a significant decrease in photocatalytic activity [119]. As suggested by Liu et al. [62] and Hong et al. [120], this decrease is a compromise between the specific surface area and the concentration ratio of surface to bulk defects in a semiconductor that results in high photocatalytic activities. In this particular case, as it was also observed by Liu et al. [62], it is the high crystallinity and, consequently, the low relative ratio of bulk to surface defects that governs the photocatalytic efficiency of the produced porous ZnO nanostructures.

In the end, the porous ZnO nanostructures calcinated at 700 °C and tested as photocatalysts were washed with de-ionized water (followed by isopropanol) and centrifuged at 4500 rpm for 5 min. After drying at 60 °C for 2 h in vacuum, these nanostructures were tested two more times for the degradation of RhB following the same experiment steps as before and keeping the same amount of powder in each run. Figure 4e shows the degradation ratio *C/C*_0_ for the three photocatalytic runs. The porous ZnO nanostructures displayed very similar performances in the photodegradation of RhB, thus demonstrating that they can be reused several times to degrade organic pollutants in water.

**Figure 5:**
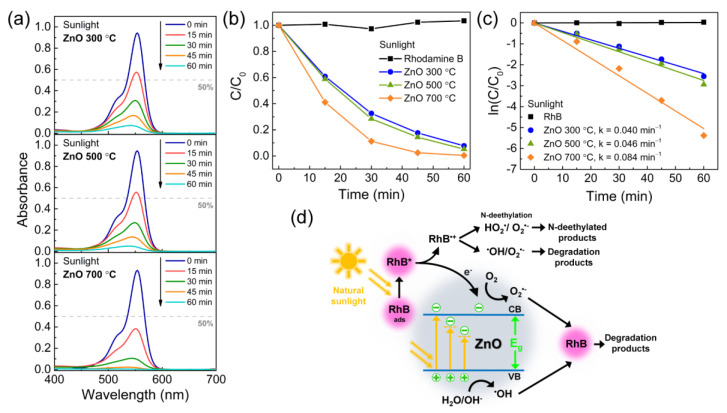
(**a**) Absorbance spectra of RhB recorded at different degradation times under solar light for ZnO samples calcinated at 300, 500, and 700 °C. (**b**) Degradation ratio *C/C*_0_ of RhB vs. sunlight exposure time for all the produced porous ZnO samples. (**c**) ln(*C/C*_0_) vs. sunlight exposure time and the resultant degradation rates *k* for each tested sample. (**d**) Solar light photocatalytic mechanism of ZnO.

Figure 5a presents the absorbance spectra of the RhB solution with the dispersed ZnO catalysts under sunlight irradiation. The photocatalytic activity of the ZnO nanostructures under sunlight is similar to the one observed under UV light. Here, RhB was also practically degraded after 60 min of sunlight exposure, with 92%, 95%, and >99% of RhB being degraded by the porous ZnO nanostructures obtained at 300, 500, and 700 °C, respectively. Figure 5b shows the degradation ratio *C/C*_0_ for the RhB solutions without and with the ZnO catalysts. From the calculated ln(*C/C*_0_) curves in Figure 5c, the degradation rate *k* was found to be slightly higher for the ZnO calcinated at 300 and 500 °C (0.040 and 0.046 min^−1^, respectively) when compared to the values determined for UV photocatalysis (0.037 and 0.038 min^−1^, respectively), whereas the *k* calculated for the ZnO produced at 700 °C (0.084 min^−1^) decreased slightly compared to its value after UV exposure (0.091 min^−1^).

Under solar light irradiation, RhB undergoes two degradation processes that occur simultaneously: a photocatalytic process, where the decomposition of the dye occurs through the destruction of its chromophore structure, and a N-deethylation process [121,122,123]. Figure 5d shows a schematic of both these processes. Like UV photocatalysis, if the semiconductor catalyst is able to absorb visible light, it will generate electron-hole (e^−^/h^+^) pairs. As explained before, the photoinduced carriers will originate ^•^OH and O_2_^•−^ radicals from the adsorbed oxygen-rich molecules on the ZnO surface, which will in turn participate in the degradation of RhB in the solution. On the other hand, it has been reported that RhB molecules can be excited by visible light [124,125]. When these molecules are adsorbed on the ZnO surface and are excited by visible light, the excited RhB* can inject electrons into the conduction band of ZnO followed by its conversion into cation radicals RhB^•+^. Adsorbed oxygen molecules can, in turn, capture the injected electrons to yield ^•^OH and O_2_^•−^ radicals. These radicals will then degrade both the created RhB^•+^ cation radicals and the RhB molecules in the solution [126]. The RhB^•+^ cation radicals can also be further decomposed into N-deethylated products by hydrolysis or by reacting with HO_2_^•^/ O_2_^•−^ radicals. The resulting N-deethylated products are responsible for a gradual hypsochromic shift in the absorbance peak since the N-deethylation of RhB is known to be a stepwise process [77,78,127,128]. This hypsochromic shift in the absorbance peak was not observed in Figure 5a, suggesting that the degradation of RhB through sunlight photocatalysis by the porous ZnO nanostructures is the predominant degradation route in these experiments.

As mentioned before, the wide bandgap energy of ZnO limits its absorption in the visible range. Nevertheless, the porous ZnO nanostructures produced at 300, 500, and 700 °C have a calculated bandgap energy of 3.25, 3.26, and 3.26 eV, respectively, and an absorption edge starting at wavelengths longer than 400 nm (see Figure 3a). This means that the ZnO nanostructures have the ability to absorb a part of the incident solar light and create e^−^/h^+^ pairs that will originate radicals for the degradation of RhB, as also demonstrated by the PL spectra obtained with different excitation wavelengths (Figure S3b). In addition, during sunlight photocatalysis, direct band-to-band excitation is much lower when compared to UV photocatalysis since solar light only comprises about 5% of UV radiation [129]. This fact suggests that under sunlight irradiation, most excitation transitions occur from the VB to energy levels within the bandgap of ZnO. Wang et al. [117] used different types of active species scavengers to study the visible photocatalytic mechanism of ZnO nanorods and found that h^+^ and ^•^OH were the main species involved in the photocatalytic degradation of RhB mediated by visible light. This finding suggests that energy levels deep inside the bandgap caused by bulk and surface defects may promote charge separation as they readily accept and trap the excited electrons, leaving behind the generated holes that will participate in the sunlight mediated degradation of RhB.

As shown by Raman and FTIR analysis, the porous ZnO structures calcinated at 300 and 500 °C have a higher quantity of defects, impurities and surface-bonded –OH that resulted from an incomplete pyrolysis of the LZHC precursor. This is also corroborated by the lower crystallinity of these nanostructures when compared to the nanostructures obtained at 700 °C (Figure 1a), which indicates a higher relative concentration ratio of bulk to surface defects. In addition, from the diffuse reflectance spectra (Figure 3a), it is clear that calcination temperatures of 300 and 500 °C yield ZnO with a higher absorption into the visible range than a temperature of 700 °C. Hence, the ZnO nanostructures calcinated at lower temperatures showed a slight improvement under sunlight irradiation when compared to UV photocatalysis due to the defect levels within the bandgap of ZnO, which promote charge generation and separation under sunlight and, subsequently, enhanced degradation of RhB.

## 4. Conclusions

In summary, porous ZnO nanostructures were successfully synthesized via a facile and fast hydrothermal method assisted by microwave irradiation followed by a calcination process at 300, 500, and 700 °C. The effect of calcination temperature on the morphological, structural, and optical properties of the porous ZnO nanostructures was investigated. The produced samples were tested as photocatalysts in the degradation of RhB under UV and natural sunlight. All porous ZnO samples showed great photocatalytic activities under UV light, with RhB being degraded within 60 min. Here, the porous ZnO obtained at 700 °C showed the best photocatalytic performance (degradation > 99%) under UV light due to its high crystallinity and consequent low concentration ratio of bulk to surface defects. Moreover, it was demonstrated that these nanostructures can be reused several times without significant changes in the photocatalytic performance. When tested under sunlight, it was verified that porous ZnO nanostructures had a similar photocatalytic performance as when tested under UV light. Here, a RhB degradation superior to 99% was also obtained when using the ZnO nanostructures calcinated at 700 °C after only 60 min of sunlight exposure, due to the ability of ZnO to absorb light in the visible range and generate e^−^/h^+^ pairs as a result of defect levels within the bandgap of this metal oxide.

## Data Availability

The data presented in this study are available on request from the corresponding author.

## Supplementary Materials

The following are available online at https://www.mdpi.com/article/10.3390/ma14092385/s1, Figure S1: Nitrogen adsorption/desorption isotherms for the porous ZnO nanostructures, Figure S2: Raman spectrum of the LZHC precursor of ZnO, Figure S3: Deconvolution of the PL spectrum of the porous ZnO nanostructures calcinated at 700 °C and PL spectra under different excitation wavelengths.
