# Supplementary material for: High UV and Sunlight Photocatalytic Performance of Porous ZnO Nanostructures Synthesized by a Facile and Fast Microwave Hydrothermal Method
Source: Materials (Basel). 2021 May 4;14(9):2385. doi: 10.3390/ma14092385 (PMC8125317; doi:10.3390/ma14092385)
Supplement: Supplementary file 1 [file materials-14-02385-s001.zip › materials-1200862-supplementary.pdf]

materials

Supplementary Materials

High UV and Sunlight Photocatalytic Performance of Porous ZnO Nanostructures Synthesized by a Facile and Fast Microwave Hydrothermal Method

Sofia Henriques Ferreira, Maria Morais, Daniela Nunes, Maria João Oliveira, Ana Rovisco, Ana Pimentel, Hugo Águas, Elvira Fortunato * and Rodrigo Martins *

i3N/CENIMAT, Department of Materials Science, Faculty of Science and Technology, Universidade NOVA de Lisboa and CEMOP/UNINOVA, Campus de Caparica, 2829-516 Caparica, Portugal; sdl.ferreira@campus.fct.unl.pt (S.H.F.); md.morais@campus.fct.unl.pt (M.M.); daniela.gomes@fct.unl.pt (D.N.); mj.oliveira@campus.fct.unl.pt (M.J.O.); a.rovisco@campus.fct.unl.pt (A.R.); acgp@campus.fct.unl.pt (A.P.); hma@fct.unl.pt (H.Á.)

* Correspondence: emf@fct.unl.pt (E.F.); rm@uninova.pt (R.M.); Tel.: +351-212948562 (E.F., R.M.)

The Supplementary Materials document contains relevant and complementary characterization data relative to the as-synthesized layered zinc hydroxide carbonate (LZHC) precursor and the porous ZnO nanostructures calcinated at different temperatures. Figure S1 presents the nitrogen adsorption/desorption isotherms for the porous ZnO nanostructures calcinated at 300, 500, and 700 °C. The Raman spectrum of the LZHC precursor is displayed in Figure S2 for comparison with the Raman spectra obtained for the porous ZnO nanostructures. To assist in the photoluminescence (PL) analysis of the obtained porous ZnO nanostructures, Figure S3 (a) shows the deconvolution of the PL spectrum of the ZnO calcinated at 700 °C into several Gaussian components and the respective values of the main gaussian peaks, whereas Figure S3 (b) depicts the PL spectra of the ZnO calcinated at 700 °C under different excitation wavelengths.

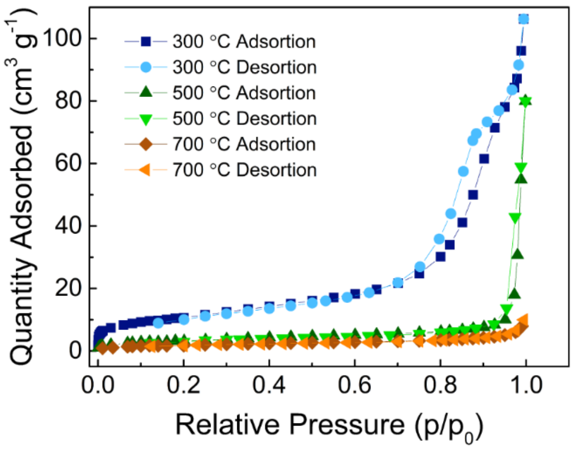

Figure S1. Nitrogen adsorption/desorption isotherms for the porous ZnO nanostructures calcinated at 300, 500, and 700 °C.

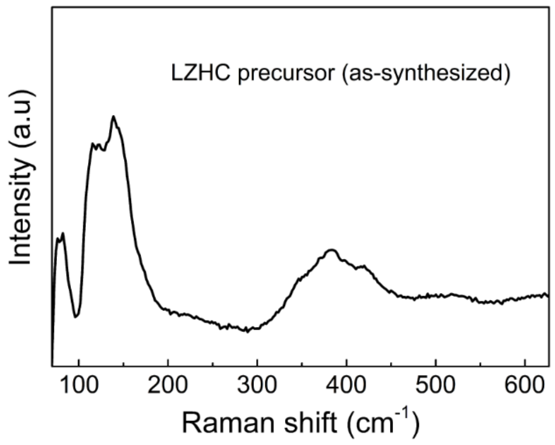

Figure S2. Raman spectrum of the LZHC precursor of ZnO, as synthesized.

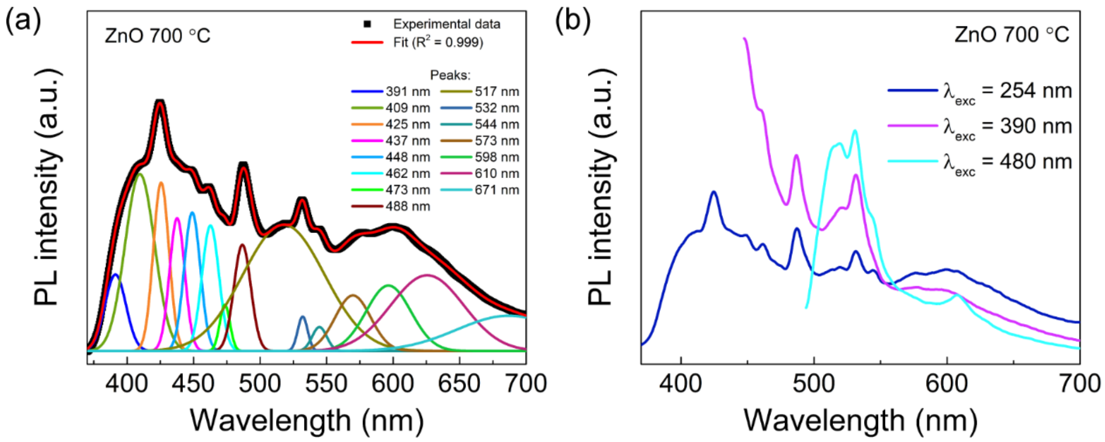

Figure S3. (**a**) Deconvolution of the PL spectrum of the porous ZnO nanostructures calcinated at 700 °C into gaussian components. (**b**) PL spectra of the porous ZnO nanostructures calcinated at 700 °C under different excitation wavelengths.
